# Pennsylvania State Veterinary Medical Association

**Published:** 1896-11

**Authors:** 


### PENNSYLVANIA STATE VETERINARY MEDICAL ASSOCIATION.

Reported for the JOURNAL by one of the staff.

THE semi-annual meeting convened at the hall of the Young Men's Christian Association, Reading, October 6, 1896, at 10.30 A.M. President Ridge called the meeting to order, and on roll-call, by Secretary Benner, the following members responded to their names: Drs. Allen, Bachman, Benner, Collins, Dreibelbis, Felton, Foelker, J. C.; Harger, Hart, John R.; Helmer, Hoskins, Houldsworth, Lusson, Michener, J. C.; Noack, Pearson, Phillips, Rayner, Thos. B.; Rayner, James B.; Reinhart, and Ridge. As delegate from the Veterinary Medical Association of New Jersey, Dr. R. C. Vail, Rahway, N. J. As visitors, Drs. A. J. Burkholder, Brackbill, Wm. P. Phipps, and Edwin Hogg.

Mayor Weidel, of Reading, was then introduced by Dr. Noack, and, in extending a welcome to the members and conferring upon the delegates in attendance the freedom of the city, he proudly referred to the rapid and substantial growth of Reading and the keen interests of her people and officials in all that goes to make up a progressive and happy community. He was not wholly unmindful of the progress and value of the veterinary profession, and was glad to note so much interest in sanitary questions. His address was responded to by member Hoskins, who, on behalf of the Association, its members, and the profession, thanked the Mayor for his warm welcome and greeting, and reminded him that by his presence alone he exhibited to the people over whom he presided a keen interest in the broadest sense for their welfare and progress. The second great problem of the day, in the great question of broad and wise municipal government, was the protection of her people from the great dangers that beset the close aggregation of large numbers of people in limited areas of territory. When the great question of a pure water-supply was well solved, there pressed on for solution the second question of world-wide importance, that of a pure food-supply; and wrapped up in this broad subject was the most important and forceful one of a pure milk-supply and a sound and wholesome meat-supply. Modern investigation in sanitary fields had revealed in part the great dangers constantly existing in these directions, and veterinary sanitary work was every day bringing forth wiser methods and better plans to deal effectively with these grave dangers, and it became the great duty of all city officials to be ever in advance of their citizens in leading in all movements to increase the safeguards from unseen dangers and thus wisely and well preserving and perpetuating to their people the greatest freedom from sickness, suffering, and disastrous results so far-reaching in every community where officials were forgetful of the great responsibilities attendant upon the honorable position of chief magistrate of a body of confiding people.

The President then read his address, which proved a thoughtful presentation of the work of an association like this. He referred to its growth and power, and how much there was dependent upon each member to fulfil his part of the labor, that there might flow forth a great return for the great privilege enjoyed. A brief review of his labors during the first period of his stewardship was referred to, and his sincere appreciation of the ready response to most of his requests that had been made upon the members. Brief outlines of his future plans were referred to with a sense of approval of the members, showing a thorough appreciation on his part of the responsibilities that followed in the train of his elevation to the Presidency. The programme was the most striking evidence of the success of his work, and it seemed a very great disappointment to all present that there was not a stronger attendance to hear the excellent papers read.

Dr. Wm. Jobson, graduate of the National Veterinary College; W. Howard Wilson, graduate of New York College of Veterinary Surgeons; Wm. P. Phipps and Edwin Hogg, graduates of the Veterinary Department of the University of Pennsylvania, were elected to membership. The resignation of Dr. A. O. Koenig was accepted. Letters of regret at inability to attend were received from Drs. Ross, Stanton, Timberman, and Rhoads.

The Corresponding Secretary's report was feelingly delivered, and the needs of the veterinary profession strongly alluded to. That their labors should be rewarded by yellow coin was startlingly elucidated, and its semi-political tenor a new innovation in scientific circles.

The Treasurer verbally reported the condition of the treasury as a healthy one, and the payment of members' dues more prompt and complete. He remarked that the collection of dues was always in order, at which suggestion there was an outpouring of dollars (mostly of the white coin) into the Corresponding Secretary's hands.

Under unfinished business the question of aiding the State Board of Veterinary Medical Examiners was discussed, and the Legislative Committee empowered to collect additional money to sustain the board in its prosecution of offenders of the several Acts governing the practice of the profession in the Keystone State. The Legislative Committee at once started a fund for this purpose, which was liberally subscribed to by those present.

Several very interesting reports were read from county secretaries, containing information of importance to the profession and some records of value to the State Board of Examiners. Reports were read and received from Berks, Crawford, Erie, Franklin, Lackawanna, Lancaster, Lebanon, Montgomery, Philadelphia, and Venango.

Chairman Zuill, of the Committee on Intelligence and Education, was absent, and for some unknown reason had failed to compile a report. Several of his co-workers present reported that they had not heard from him, and, therefore, were unable to make any response to this important aspect of association work.

The report of the Committee on Sanitary Science and Police was called for, and Chairman Pearson responded in his usual interesting and felicitous manner, apologizing first for his inability, for want of time, to present a carefully written report, after which he immediately plunged into the text of his subject, not forgetting to give the JOURNAL a vigorous shot at its editorial destined to allay the fear of the people and thus lessen the dangers of rabies and hydrophobia. He reported an extremely interesting infectious venereal disease that had prevailed in enzoötic form near Brookville, Jefferson County. It was found to exist in an imported French black stallion and thirteen of the mares he had covered. It was accompanied with urethritis and ulcers of the penis in the horse, and in the mares by a vesicular eruption of the vulva and vagina, with purulent discharges. The stallion subsequently died and was reported as having broken-out with sores over the body like farcy before death. The stallion was quarantined for some months before his death, and the mares were restricted to the farms where they belonged. The disease was not el dourine, but, as diagnosed by Dr. Pearson, a contagious vesicular disease. Under creolin and sulphate of zinc solutions the mares made good recoveries. The stallion covered successfully only one of the mares. A number of reports of anthrax were received from the mountainous districts, where boggy areas existed. In many cases reported it proved to be red-water from the eating of weeds and over territory that had been burned the year before. Thirty-five cases of glanders were reported. Rabies and its correlative, hydrophobia, had proven of much concern and created quite a scare. Investigations were conducted in Union, Centre, and other counties. In Union County a collie dog was at large for two days with rabies, biting many other dogs, all of which were killed save one, which escaped to Centre County, where it attacked a steer, other dogs, and pigs, which became rabid. Another rabid dog, at large for three weeks, ran west and entered Penn Valley, where it bit many dogs, some swine, involving seven farms. On one farm fourteen out of fifteen swine were bitten. There were five propagations of the disease in three months. In Columbia County, Dr. Winner, a veterinary surgeon, of Bloomsburg, was bitten, which in three weeks was followed by paralysis of the throat, mania, and death. In Allegheny County two men and three boys had died with hydrophobia. Another case, sent three weeks after being bitten to Pasteur Institute, New York, was so ill when Harrisburg was reached that he was taken off the car in spasms and died next day. Hog-cholera has prevailed in several sections, 300 to 400 having died in Centre County. Wherever prevalent the premises were disinfected and herds quarantined. As to tuberculosis, the State Veterinary Sanitary Board had received severe criticism for its extreme conservatism in dealing with the disease. Some 2400 animals had already been examined, of which 25 per cent. were tuberculous and destroyed, involving an expense of $14,000. In some parts of the State but very few cases were found; of Lackawanna County this was true. In Bradford, the great dairy county, the disease has been found in but two herds, both of these of registered animals. In one herd of 73, 59 were tuberculous; in another herd of 12, 8 were tuberculous; most herds were free. Five or six different veterinarians were employed, and three different preparations of tuberculin used. Reports of cerebro-spinal meningitis were received, but no action taken. The resolutions of this Association adopted in March have terminated in the Board allotting money for a laboratory at the Veterinary Department in Philadelphia, and the employing of a bacteriologist to investigate infectious abortion, cerebro-spinal meningitis, osteoporosis, etc. Another disease affecting a considerable number of cattle and first thought to be tuberculosis, and where tuberculin was used, failed to give any reactions, proved, on killing the animals, to be a peculiar form of pneumonia—a chronic pneumonia with greatly increased fibrous tissue. A section showed areas of necrosis resembling tuberculosis. Inoculation of other animals failed to produce any tuberculosis, and other tests were equally negative. One other similar outbreak was reported through the Bureau of Animal Industry, in the stock-yards of Baltimore, and one in Adams County, of this State, where two or three herds were found affected, probably due to some local conditions. Diagnosis—chronic catarrhal pneumonia. The poultry interests of Pennsylvania are a very valuable and important industry, 9,000,000 in number produced in one year $14,000,000 of eggs. The poultry-yards of our country are as valuable as our wheat-crop. The poultry-yards of Pennsylvania are worth as much nearly as our dairy products. Ten per cent. of these fowls die annually. The diseases of poultry will be referred to in an early bulletin to be issued by the State Live-stock Sanitary Board. The report of the Chairman awakened the most intense interest and was highly appreciated.

The reading of papers being in order, the President called upon Dr. Hart, who informed the convention that he was under the impression that he was to read at the annual meeting. Dr. J. C. Michener then offered another of his thoroughly practical presentations, bearing upon "Parturient Apoplexy,"[1] with a record of the last seventeen cases treated. It was full of strong practical aspects and measures of treatment, and enjoyed by all. Dr. Francis S. Allen then presented a well-prepared paper on "Acetanilid,"[2] with numerous diagrams showing its results in high temperatures. The paper exhibited great care in its preparation and records of personal experience with the drug of much value. Dr. James T. Ross's paper on the "Management of Parturient Apoplexy Homœopathically" was read by the Secretary, and was an additional contribution to the widely adopted general plan of the administration of small doses of aconite and belladonna alternately. Dr. Howard B. Felton presented the subject of "Canine Distemper"[3] in a masterly manner, a wide personal experience with special opportunities giving him unusual opportunities to test the value of the various plans of treatment advocated. It proved a paper of great merit and value, and will be read by everyone interested in canine pathology with great benefit and appreciation. Dr. Jacob Helmer read a thoughtfully prepared and convincing presentation of "Homœopathy and Why I Do Not Practise It."[4] It was a severe arraignment of this method of medical treatment based upon earnest study, a period of practical experience in its application, and a thorough and deep conviction of its false promises and absurd claims. It will be read by all with much interest, and will strongly

[1] See December number.

[2] See December number.

[3] See page 769.

[4] See page 759.

sustain those who have adhered to the regular lines of practice, accepting all good and reasonable scientific plans for the alleviation of disease, and will surely shake up severely those who from time to time have superficially drifted into lines of homœopathic treatment. Dr. W. Horace Hoskins presented briefly the uses of "Pyoktanin," and reported the experiences of a number of veterinarians who had used it in practice. The many interesting reports and papers had consumed so much time that when the hour of adjournment was reached there was no time left for discussion of the same. Vice-President James B. Rayner being called to the chair, delegate Vail, of New Jersey, presented for information the subject of "Paralysis of the Pharynx and its Closely Allied Condition to Cerebro-spinal Meningitis," on which a few members spoke briefly, when a motion for adjournment prevailed.

